# Why Do People Die Too Young?

**Published:** 1923-02

**Authors:** F. G. Soule

**Affiliations:** President National Bureau of Analysis, Chicago


### WHY DO PEOPLE DIE TOO YOUNG?

BY F. G. SOULE, PRESIDENT NATIONAL BUREAU OF ANALYSIS, CHICAGO

This was the question forever presenting itself to me during the twelve years I was manager of a life insurance company.

Life insurance is all right for the comfort and pleasure of others after you are dead. But my ambition was to work out a practical plan of "insurance" that would keep men and women above ground and share in those comforts and pleasures themselves.

The question occupied my mind day and night. I had long noticed the increasing number of men and women, apparently in robust health, whose insurance examinations had shown albumin or sugar in the urine. To the insurance companies this suggested Bright's disease or diabetes, and as these insidious diseases are the causes of most of insurance losses the applicants had been promptly rejected.

I found that most of these applicants had gone, year after year, without the slightest knowledge of the condition of their kidneys, liver or digestive organs—the most delicate and yet most overworked organs of the body. And fully sixty per cent. of them had passed every health test except the urinalysis. There they had been found to be below par.

"There must be a way," I thought, "to discover these conditions when they first appear—some way of giving timely notice when these organs begin to go wrong so that the tendency to trouble can be corrected before it runs into actual disease."

The more I studied the subject the more interesting it became. I consulted physicians, our own staff of doctors, specialists, and general practitioners. Wherever I turned the same answer met me: "The health of the human body is shown principally by the condition of the kidneys as revealed in the urine by chemical and microscopical analysis."

One physician of great eminence put it before me so clearly that his words are still fresh in my mind. He said: "Every drop of blood in our bodies passes through the kidneys once every seven minutes. The impurities of the blood are eliminated by the kidneys and when the work of eliminating these waste products produces a structural change in the kidneys, such condition is revealed through the urinalysis. If more people realized this fact and knew how easy it is to correct improper conditions by taking them in hand early there would be fewer deaths and a much healthier race."

Indeed the claim of the French scientists seemed well founded, that, "as the kidneys are, so are all the organs of the body."

But what encouraged me most was to learn that these diseases that end so fatally are usually preceded by derangements of the intestinal digestion. As the great

French authority, Charcot, put it: "Ninety per cent. of all diseases originate in the intestinal tract."

I say that I was encouraged, especially when I learned that these "intestinal derangements" are really the timely warnings of Nature—revealed by scientific examinations of the urine—and, when thus discovered, may be easily corrected by care in diet.

And even when these examinations had been neglected, and, for lack of timely information, the intestinal troubles had run into chronic Bright's disease or diabetes, I found that even these insidious diseases themselves could be controlled to a surprising degree by intelligent suggestions as to diet.

Here, then, was the answer to the question—right in my own business—in my own office.

I worked for months perfecting my service. They were anxious months because I knew the service must establish a reputation for the highest accuracy; that it must be absolutely convenient to the subscriber, taking no time from his business or he would neglect it; and that the reports must be so plain as to be easily understood.

My next step was to select twenty-five leading men of Chicago and acquaint them with my plans. Without exception they assured me of their support. Feeling confident of the great good I could accomplish and with the co-operation of these able men I started my service which was the first periodical health checking service ever started in any land.

That was twelve years ago. Today the thousands of my subscribers in all parts of America and Canada—without giving it more than four minutes of time a year—are able to have periodical soundings taken of their state of health. And in the past twelve years, during which this modern method of reclaiming years of life has been in operation, hundreds have been saved from premature death by being warned in time of improper physical conditions of which they were not aware.

Life is just as precious to you and to your dear ones as it is to the biggest millionaire in the world. You are just as anxious to live long and well as any man. Your life is perhaps more important to your dear ones than the life of the very rich man to his family, for your dear ones may be entirely dependent on your being well and strong to work for them.

Even if you are personally indifferent as to your own tenure of life, there is a great duty you owe to your family. Those who depend on you for their support need you. They need your work. They need the comfort and happiness of your presence. They need your strong protecting arm.

You may look well, feel well, and have all the evidence of health, yet be far from actual health. Some of the worst wasting diseases show no outward sign of their ravages until it is too late to halt them. These are the very ailments and conditions that we may be able to detect in time for them to be corrected by the simple means suggested in our reports.

If you are sick and know you are sick—by all means consult your physician. If you are well and happy, write for our booklet, "Why People Die Too Young," and let us show you how to keep well and happy.

